# Protein cysteine S-nitrosylation provides reducing power by enhancing lactate dehydrogenase activity in *Trichomonas vaginalis* under iron deficiency

**DOI:** 10.1186/s13071-020-04355-0

**Published:** 2020-09-18

**Authors:** Wei-Hung Cheng, Kuo-Yang Huang, Seow-Chin Ong, Fu-Man Ku, Po-Jung Huang, Chi-Ching Lee, Yuan-Ming Yeh, Rose Lin, Cheng-Hsun Chiu, Petrus Tang

**Affiliations:** 1 Department of Parasitology, College of Medicine, Chang Gung University, Guishan District, Taoyuan City, Taiwan; 2 Graduate Institute of Pathology and Parasitology, National Defense Medical Center, Taipei, Taiwan; 3 Department of Biomedical Sciences, College of Medicine, Chang Gung University, Guishan District, Taoyuan City, Taiwan; 4 Genomic Medicine Core Laboratory, Chang Gung Memorial Hospital, Linkou, Taiwan; 5 Department of Computer Science and Information Engineering, College of Engineering, Chang Gung University, Guishan District, Taoyuan City, Taiwan; 6 Molecular Infectious Disease Research Center, Chang Gung Memorial Hospital, Linkou, Taiwan

**Keywords:** *Trichomonas vaginalis*, Iron deficiency, Glycolysis, Cysteine S-nitrosylation, Lactate dehydrogenase, Nicotinamide adenine dinucleotide

## Abstract

**Background:**

Iron plays essential roles in the pathogenesis and proliferation of *Trichomonas vaginalis*, the causative agent of the most prevalent non-viral human sexually transmitted infection. We previously demonstrated that under iron deficiency, the endogenous nitric oxide (NO) is accumulated and capable of regulating the survival of *T. vaginalis*. Herein, we aim to explore the influence of NO on the activity of the pyruvate-reducing enzyme lactate dehydrogenase in *T. vaginalis* (TvLDH).

**Methods:**

Levels of lactate and pyruvate were detected for determining glycolysis activity in *T. vaginalis* under iron deficiency. Quantitative PCR was performed to determine the expression of TvLDH. S-nitrosylated (SNO) proteomics was conducted to identify the NO-modified proteins. The activities of glyceraldehyde-3-phosphate dehydrogenase (TvGAPDH) and TvLDH were measured after sodium nitrate treatment. The effects of protein nitrosylation on the production of cellular reducing power were examined by measuring the amount of nicotinamide adenine dinucleotide (NAD) and the ratio of the NAD redox pair (NAD^+^/NADH).

**Results:**

We found that although the glycolytic pathway was activated in cells under iron depletion, the level of pyruvate was decreased due to the increased level of TvLDH. By analyzing the SNO proteome of *T. vaginalis* upon iron deficiency, we found that TvLDH is one of the glycolytic enzymes modified by SNO. The production of pyruvate was significantly reduced after nitrate treatment, indicating that protein nitrosylation accelerated the consumption of pyruvate by increasing TvLDH activity. Nitrate treatment also induced NAD oxidation, suggesting that protein nitrosylation was the key posttranslational modification controlling cellular redox status.

**Conclusions:**

We demonstrated that NO-mediated protein nitrosylation plays pivotal roles in the regulation of glycolysis, pyruvate metabolism, and the activity of TvLDH. The recycling of oxidized NAD catalyzed by TvLDH provided the reducing power that allowed *T. vaginalis* to adapt to the iron-deficient environment.
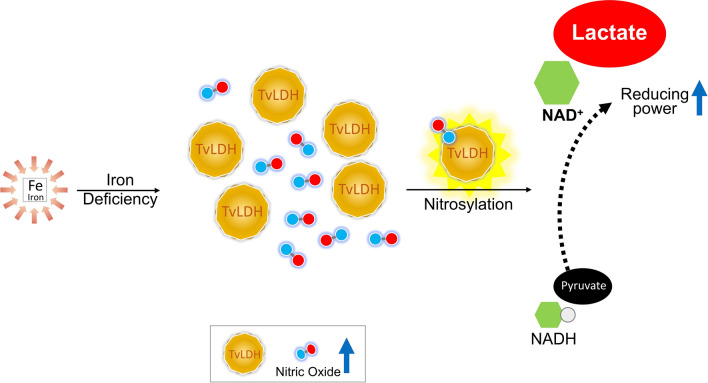

## Background

Trichomoniasis is the most common sexually transmitted infection (STI) of non-viral origin in humans and is caused by the parasitic protozoan *Trichomonas vaginalis* [1, 2]. *Trichomonas vaginalis* resides in the human urogenital tract and may be asymptomatic or cause a variety of clinical features in both genders, including severe inflammation. Although trichomoniasis is usually self-limiting in humans, it increases the risks of prostate and cervical cancer, pelvic inflammatory disease, infertility, and HIV transmission [3, 4]. Trichomoniasis has become a more severe public health issue because of increasing treatment failure. Thus, seeking new strategies for the treatment and prevention of this infection should be prioritized.

Glucose is the major carbon resource for *T. vaginalis* [5]. In general, pyruvate, the end product of glycolysis, is converted to acetyl-CoA by pyruvate:ferredoxin oxidoreductase (PFO) in the hydrogenosome [6]. Under iron deficiency, *T. vaginalis* exhibits a slow-growing or arrested-growth phenotype since the protist utilizes iron-dependent metabolic systems to generate energy and other acetyl-CoA-related compounds [7]. In mammals, the expression of mitochondrial tricarboxylic acid (TCA) cycle enzymes, such as aconitase and succinate dehydrogenase, is positively regulated by iron [8, 9]. The inhibition of energy production caused by iron deficiency can be compensated by more active glycolysis [10]. The hydrogenosomal energy metabolism of *T. vaginalis* is downregulated by iron shortage; however, the regulation of glycolysis associated with iron availability remains unclear [7]. For this parasite, residing in an environment with continuously fluctuating iron availability, mechanisms to respond to iron limitation are important for its adaptation and survival.

Nicotinamide adenine dinucleotide (NAD) serves as the prosthetic group in several metabolic reactions, including glycolysis and fatty acid β-oxidation because of its electron acceptor and donor characteristics [11]. Additionally, sirtuin and poly (ADP-ribose) polymerase (PARP) use the oxidized form of NAD (NAD^+^) as a cofactor for their enzymatic reactions, thereby regulating gene expression and influencing chromatin structure and DNA repair [12, 13]. Maintenance of NAD level and the ratio of NAD^+^ to NADH is important for ensuring the metabolic directions and functionality of NAD^+^-dependent enzymatic reactions [14]. Thus, the recycling of NAD^+^/NADH is a critical step for maintaining cellular metabolic status. The regeneration of NAD^+^ from NADH relies on the activity of lactate dehydrogenase (LDH) [15]. According to our previous transcriptomics analysis [16], TvLDH is one of the most upregulated genes upon iron deficiency. However, the role of TvLDH in the regulation of NAD in *T. vaginalis* is poorly understood.

Our previous report showed that NO is crucial for maintaining the viability of *T. vaginalis* upon iron deficiency. NO is generated *via* nitric oxide synthase (NOS), which converts arginine to NO and citrulline. *T. vaginalis* does not possess NOS; however, the protist uses arginine as the substrate for ~ 30% NO production *via* an unknown mechanism [16]. Although NO accumulation causes cellular damage, it also has beneficial effects on cells. For instance, increased NO levels trigger the production of antioxidants that protects cells from oxidative stress [17]. Tyrosine nitration and cysteine S-nitrosylation of proteins are key posttranslational modifications (PTMs) mediated by oxidized forms of NO, i.e. nitrate and nitrite. Recent profiles of S-nitrosylated and nitrated proteomes in protists showed that glycolytic enzymes and ribosomal proteins are the general targets for SNO modification [18–21]. It has been reported that protein nitrosylation enhances the activity of glycolytic enzymes in mice [22]. These observations suggest that glucose metabolism is possibly regulated by NO-dependent PTMs in protists.

*Trichomonas vaginalis* encounters continuously changing iron levels in the vagina; however, the mechanisms underlying its adaptation to iron fluctuation are not well documented. In this study, we performed SNO proteomics to illustrate the relationships between NO and glycolytic enzymes in *T. vaginalis* upon iron deficiency. The findings suggest that TvLDH plays a key role in providing oxidized NAD (NAD^+^) to maintain the balance of cellular redox homeostasis.

## Methods

### *Trichomonas vaginalis* and treatments

*Trichomonas vaginalis* (ATCC_30236) was used in this study. An iron-deficient (ID) group of *T. vaginalis* was established by the addition of 180 M dipyridyl (DIP; Sigma-Aldrich, Merck, Germany) to yeast extract, iron-serum (YI-S) medium at a density of ~ 1 × 10^6^/ml, and an iron-rich (IR) cell group was established using YI-S medium containing ferrous ammonium citrate (FAC) [16]. To create a nitrate treatment for the ID cells, sodium nitrate (25 mM, Sigma-Aldrich) was added at the same time DIP was added. The viability of trichomonad cells in the different treatments was assessed by trypan blue exclusion assay using a hemocytometer (Reichert Technologies, Depew, NY, USA).

### Sequence analysis and expression profiles of TvLDHs

The protein sequences of putative LDH and MDH orthologs were collected from TrichDB [23]. The TvLDH-specific leucine 91 was used as an indicator for annotating TvLDHs [24]. TvLDHs and TvMDHs were aligned by T-coffee, and the identity among the proteins were estimated by using the NCBI BLAST tool [25]. The expression patterns of TvLDH short reads derived from next-generation sequencing were extracted from previous mapping results [16].

### RNA extraction and quantitative reverse transcription polymerase chain reaction

The total RNA of *T. vaginalis* cultured under iron-rich and -deficient conditions was extracted for determining the expression levels of TvLDHs [16]. Briefly, the cell pellets were resuspended in TRI Reagent Solution (Invitrogen, Thermo Fisher Scientific, Waltham, MA, USA) and incubated at RT for 5 min. After adding chloroform, the mixture was incubated at room temperature for 15 min. The RNA fraction was generated by 16,750×*g* centrifugation at 4 °C for 15 min and collected. Diethylpyrocarbonate (DEPC)-treated 70% alcohol was added to wash the RNA pellets, and the air-dried pellets were reconstituted in DEPC-treated water.

cDNA corresponding to each condition was generated from mRNA *via* reverse transcription. The mRNA was supplemented with oligo-dT primer and dNTPs (Invitrogen, Thermo Fisher Scientific) and incubated at 65 °C for 5 min. A cDNA synthesis mix containing ThermoScript^TM^ III reverse transcriptase (Invitrogen, Thermo Fisher Scientific), RNaseOUT (Promega, Madison, WI, USA), and dithiothreitol (Sigma-Aldrich, Merck) was added to the mixture. cDNA conversion was performed *via* a series of incubations (25, 50, and 85 °C for 5, 60, and 15 min, respectively). RNA was removed from the RNA-cDNA hybrids *via* RNase H treatment at 37 °C for 20 min.

The cDNA was then subjected to real-time polymerase chain reaction (real-time PCR). Ribosomal protein L8 (TVAG_104490) was used as the internal control for data normalization (forward primer: 5′-TTG CGG TAT CAA GAT GAA CCC AG-3′, reverse primer: 5′-GAA CCA AAG CTT TAT GCA AGG TGA-3′) [16]. The reaction mixture contained cDNA, TOOLS 2× SYBR qPCR mix (Biotools, New Taipei City, Taiwan), and specific primer sets (Additional file 1: Table S1), and the reaction was performed using a QuantStudio 3 instrument (Applied Biosystems, Thermo Fisher Scientific).

### Lactate and pyruvate measurements

Lactate and pyruvate levels in trichomonad cells were determined according to the manufacturer’s instructions (Lactate Colorimetric Assay Kit II (K627) and Pyruvate Colorimetric/Fluorometric Assay Kit (K609; BioVision, Milpitas, CA, USA). Briefly, ~ 1 × 10^6^ cells per sample were collected and washed twice with cold-PBS. The cell pellets were resuspended in Assay Buffer and incubated on ice for 10 min. The cell debris was precipitated by high-speed centrifugation, and the supernatant was collected for analysis. The standard and reaction mixes for these assays were prepared as recommended by the manufacturer. Each sample and standard (50 µl) was loaded onto a 96-well ELISA plate, to which 50 µl of reaction mix was added. After incubation for 30 min at RT, the absorbance at 450 and 570 nm was recorded by an ELISA reader (SpectraMax M2e; Molecular Devices, San Jose, CA, USA) for the lactate and pyruvate assay, respectively.

### Biotin switch assay and SNO protein purification

The S-nitrosylated proteins were visualized by biotin-switch assay following the manufacturer’s guidelines (S-Nitrosylated Protein Detection Kit (Biotin Switch), Item No. 10006518, Cayman Chemical, Ann Arbor, MI, USA) [19]. Cells were washed twice with Wash Buffer. The pellets were resuspended in “Buffer A containing Blocking Reagent” and incubated for 30 min at 4 °C with shaking. The incubated samples were centrifuged at 130,000×*rpm* for 10 min at 4 °C, and the supernatant was transferred to 15 ml centrifuge tubes. Two milliliters of ice-cold acetone was added to each sample, and the mixture was incubated at − 20 °C for at least 1 h. The protein of each sample was pelleted by centrifugation for 10 min at 4 °C. “Buffer B containing Reducing and Labeling Reagents” was added to resuspend the proteins, with incubation for 1 h at room temperature. The biotinylated protein was precipitated by acetone as described above and rehydrated with the appropriate amount of Wash Buffer.

The biotin-labeled proteins were next captured by streptavidin-conjugated beads. Following the manufacturer’s instructions, streptavidin-coupled magnetic beads (GE Healthcare, Merck, Germany) were equilibrated with binding buffer (Tris-buffered saline, TBS) before adding the biotinylated proteins. The protein-beads mixture was incubated for 30 min at 4 °C with gentle shaking. The unbound proteins were removed by washing with 2 M urea-containing TBS. The biotinylated proteins were then transferred to a new tube for trypsin digestion.

### Western blotting

The tyrosine-nitrated and biotinylated proteins were assessed by the streptavidin-horseradish peroxidase (HRP) approach. Briefly, proteins were separated by sodium dodecyl sulfate polyacrylamide gel electrophoresis (SDS-PAGE) and transferred to a nitrocellulose membrane. The membrane was blocked with 3% bovine serum albumin (BSA) in Tris-buffered saline Tween-20 (TTBS) overnight. For the detection of biotinylated proteins, S-Nitrosylation Detection Reagent was used at 1:5000 dilution and incubated for 1 h at room temperature. Anti-nitrotyrosine antibody (1:500, Merck) was added to the blocking buffer, and the membrane was incubated overnight with gentle shaking. Secondary antibody coupled with HRP (BioTools) was added to the membrane, which was incubated at RT for 40 min. After washing steps, the membrane was developed with enhanced chemiluminescence (ECL) substrate and visualized using a Gel Doc imaging system (Bio-Rad).

### In-solution digestion

The biotin-labeled products were eluted by addition of 0.15% trifluoroacetic acid (TFA), vacuum dried and reconstituted in 50 mM ammonium bicarbonate (ABC) and then reduced with 10 mM dithiothreitol (DTT, Sigma-Aldrich, Merck) at 56 °C for 45 min. Next, cysteine blocking was performed with 40 mM iodoacetamide (IAM, Sigma-Aldrich, Merck) at 25 °C for 30 min. The samples were digested with sequencing-grade modified porcine trypsin (Promega) at 37 °C for 16 h. The peptides were then desalted, dried by vacuum centrifugation, and stored at − 80 °C until use.

### LC-MS analysis and protein identification

The dried peptide mixtures were reconstituted in HPLC buffer A (0.1% formic acid, Sigma-Aldrich, Merck) and loaded onto a reverse-phase column (Zorbax 300SB-C18, 0.3 × 5 mm; Agilent Technologies, Santa Clara, CA, USA). The desalted peptides were then separated on a homemade column (HydroRP 2.5 μm, 75 μm I.D. × 20 cm with a 15 μm tip) using a multistep gradient of HPLC buffer B (99.9% acetonitrile/0.1% formic acid) for 70 min with a flow rate of 0.25 μl/min. The LC apparatus was coupled to a 2D linear ion trap mass spectrometer (Orbitrap Elite ETD; Thermo Fisher Scientific) operated using Xcalibur 2.2 software (Thermo Fisher Scientific). Full-scan MS was performed in the Orbitrap over a range of 400 to 2000 Da and a resolution of 120,000 at m/z 400. Internal calibration was performed using the ion signal of [Si(CH3)2O]6H+ at m/z 536.165365 as lock mass. The 20 data-dependent MS/MS scan events were followed by one MS scan for the 20 most abundant precursor ions in the preview MS scan. The m/z values selected for MS/MS were dynamically excluded for 40 s with a relative mass window of 15 ppm. The electrospray voltage was set to 2.0 kV, and the temperature of the capillary was set to 200 °C. MS and MS/ MS automatic gain control were set to 1000 ms (full scan) and 200 ms (MS/MS), or 3 × 10^6^ ions (full scan) and 3 × 10^3^ ions (MS/MS) for maximum accumulated time or ions, respectively.

The data analysis was carried out using Proteome Discoverer software (version 1.4, Thermo Fisher Scientific). The MS/MS spectra were searched against the UniProt database (extracted for *T. vaginalis*, 50,827 sequences) using the Mascot search engine (Matrix Science, London, UK; version 2.5). For peptide identification, 10 ppm mass tolerance was permitted for intact peptide masses, and 0.5 Da was permitted for CID fragment ions with allowance for two missed cleavage sites from the trypsin digestion. oxidized methionine and acetyl (protein N-terminal) were set as variable modifications, and carbamidomethyl (cysteine) was set as a fixed modification. Peptide-spectrum matches (PSMs) were then filtered based on high confidence and a Mascot search engine rank of 1 for peptide identification to ensure an overall false discovery rate below 0.01. Proteins with only a single peptide hit were removed.

### GAPDH activity assays

The activity of TvGAPDH was measured as described by the manufacturer (GAPDH Activity Assay Kit (K680), BioVision). The cells (~ 10^6^) of each condition were collected, washed with cold-PBS, and lysed by Assay Buffer. After high-speed centrifugation, the supernatant was collected and loaded into the testing wells. The reaction mix (GAPDH Developer and Substrate) was added to the samples, which were incubated at 37 °C for 1 h. The absorbance of each sample at 450 nm was measured every 10 min, and the NADH concentration was determined according to a standard curve. The activity of GAPDH in each sample was calculated and expressed as nmol/min/μl.

### LDH activity assay

The activity of TvLDH after sodium nitrate treatment was determined according to the manufacturer’s instructions (Lactate Dehydrogenase Activity Assay Kit (MAK066), Sigma-Aldrich, Merck). Cells (~ 10^6^) of iron-deficient and sodium nitrate-treated groups were collected, washed with cold-PBS, and lysed with Assay Buffer. The supernatant was collected following high-speed centrifugation and added to wells for analysis. The Substrate Mix solution was added to the samples, which were then incubated for 30 min. The absorbance of each sample at 450 nm was detected every 5 min, and the NADH concentration was determined according to a standard curve. The activity of LDH in samples was calculated and expressed as mU/ml.

### NAD^+^/NADH assay

Total NAD and the NAD^+^ to NADH ratio were examined following the manual provided by the manufacturer (NAD/ NADH Quantitation Colorimetric Kit (K337), BioVision). The cells (4 × 10^6^) were collected and washed with cold-PBS. The cell pellets were resuspended by the addition of Extraction Buffer and lysed by freeze/thaw cycles. The deproteinized supernatant was separated into two parts for measurements: total NAD^+^ with NADH and NADH only (NAD^+^ decomposition was performed *via* 60 °C incubation for 30 min). The reaction mix (NAD Cycling Buffer and Enzyme Mix) was added to the wells along with the samples, and the samples were incubated at RT for 5 min. NADH developer was then added to the wells, and the samples were again incubated at RT for 2 h. The absorbance of each well at 450 nm was measured, and the concentration was calculated according to a standard curve. The NAD^+^ amount was determined by subtracting the amount of NADH from the total amount of NAD^+^ and NADH.

### Statistical analysis

Student’s t-tests were used to analyze the data derived from biological repeats using GraphPad Prism 5 software. Asterisks denote test significance according to *P*-value: * *P* < 0.05, **P < 0.01, and *** *P* < 0.001.

## Results

### Pyruvate reduction, lactate accumulation, and TvLDH upregulation were induced by iron deficiency

**Fig. 1:**
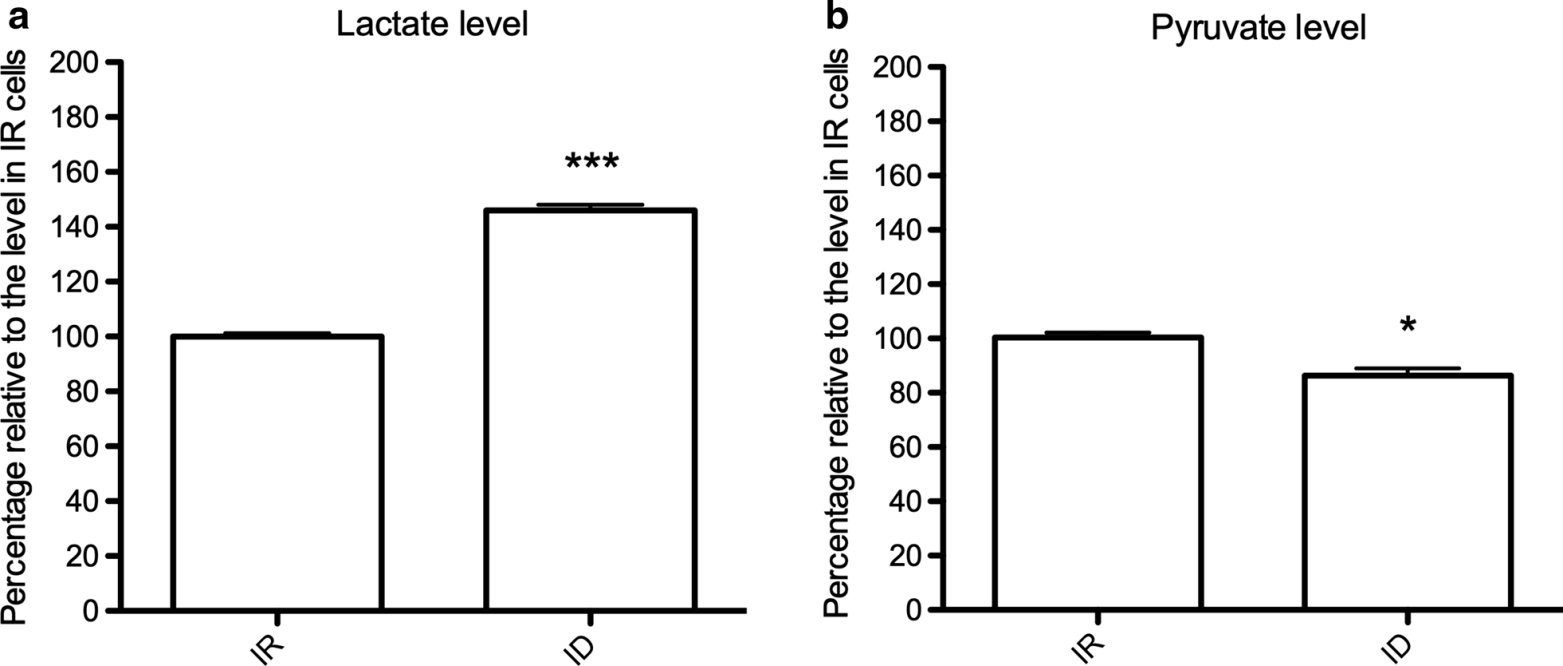
Glycolysis of *T. vaginalis* was induced by iron deficiency. Lactate and pyruvate levels were measured to monitor the glycolysis efficiency of trichomonad cells cultured under different levels of iron availability. **a** The lactate level of the iron-deficient group (ID, 180 µM DIP) relative to that of the iron-rich control group (IR, 80 µM FAC) is shown. *** *P* < 0.001 compared with the IR group. **b** The pyruvate level of the ID group relative to that of the IR control group is shown. The data are presented as the mean ± SD of three independent experiments. * *P* < 0.05 compared with the IR group

Glycolysis is regulated by iron concentration in mammals; however, limited information regarding this process in *T. vaginalis* is available [10, 26]. Hydrogenosomal energy metabolism was found to be significantly reduced by iron limitation, suggesting that enhanced glycolysis may be a compensatory mechanism for energy production in *T. vaginalis* [7]. In our previous RNA-Seq analysis, the expression of glycolytic enzymes did not differ between iron-rich (IR) and iron-depleted (ID) conditions (Additional file 2: Table S2) [16]. To clarify whether glycolysis is affected by iron availability, lactate production was measured following previous studies [27, 28]. The amount of lactate in ID cells was ~ 150% of the amount in IR cells, indicating that glycolysis was more active under iron deficiency (Fig. 1a). Pyruvate is an intermediate in multiple metabolic pathways. To reveal whether pyruvate accumulates or is consumed in response to active glycolysis under ID conditions, we detected the amount of pyruvate in *T. vaginalis* cultured under different iron concentrations. As shown in Fig. 1b, the amount of pyruvate was lower in the ID treatment, implying that pyruvate was converted into downstream compounds, such as acetyl-CoA, alanine and lactate.

**Fig. 2:**
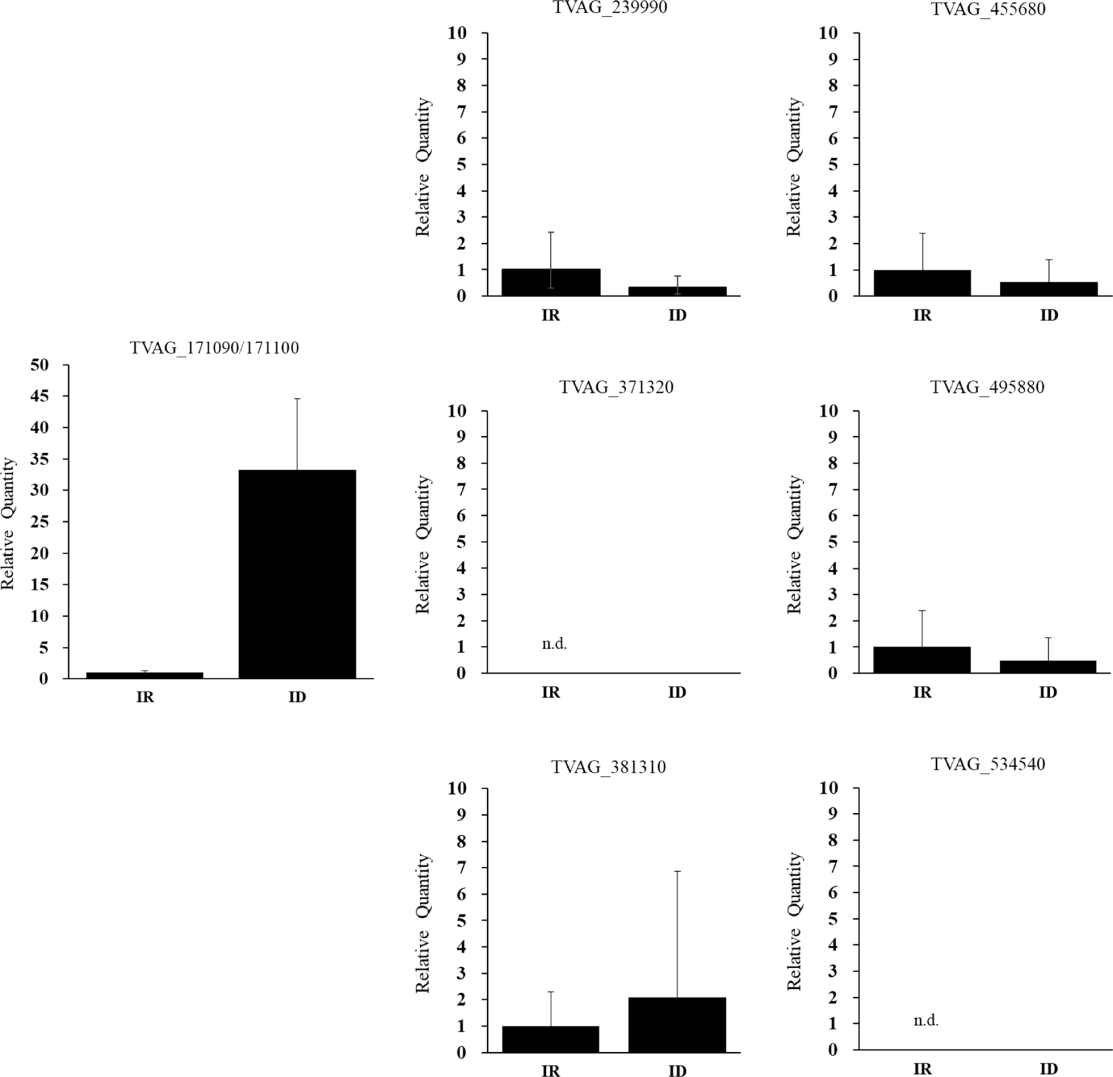
The expression of TvLDH (TVAG_171090) was upregulated under iron depletion. The mRNA expression levels of TvLDHs were detected by real-time PCR with specific primer sets. The expression patterns of TvLDHs in the iron-deficient group (ID) relative to the iron-rich control group (IR) are shown

Our previous transcriptomics analysis showed that TvLDH (TVAG_171090) was greatly upregulated upon iron depletion [16]. Due to high sequence similarity between TvLDHs and malate dehydrogenases (TvMDHs), we filtered these protein sequences with the key amino acid residue leucine 91 (L91) [24]. All TvMDH and TvLDH protein sequences were aligned, and a total of 8 TvLDHs were categorized by the presence of L91 (Additional file 3: Alignment S1). We performed quantitative PCR to assess the relative levels of TvLDHs under different levels of iron availability (Fig. 2). The expression level of TVAG_171090 was ~ 30-fold higher in *T. vaginalis* cultured under ID conditions than in those cultured under IR conditions, consistent with our previous RNA-Seq data. The open reading frames of TVAG_171090 and TVAG_171100 were undistinguishable; for this reason, primers for the individual genes could not be designed. The number of unique reads mapped to TVAG_171090 was much greater than that mapped to TVAG_171100 because of the annotated sequence of 3′-untranslated regions (Additional file 4: Figure S1). These results suggested that TVAG_171090 was the dominant ortholog expressed in *T. vaginalis* under ID treatment.

Although the expression of glycolytic enzymes was not significantly altered by iron depletion, more active glycolysis might be regulated by post-translational mechanisms. Moreover, whether upregulated TvLDH leads the metabolic direction of pyruvate should be investigated to further reveal the role of glycolysis in the response to iron-deficiency stress.

### Glycolytic enzymes were modified by protein nitrosylation

**Fig. 3:**
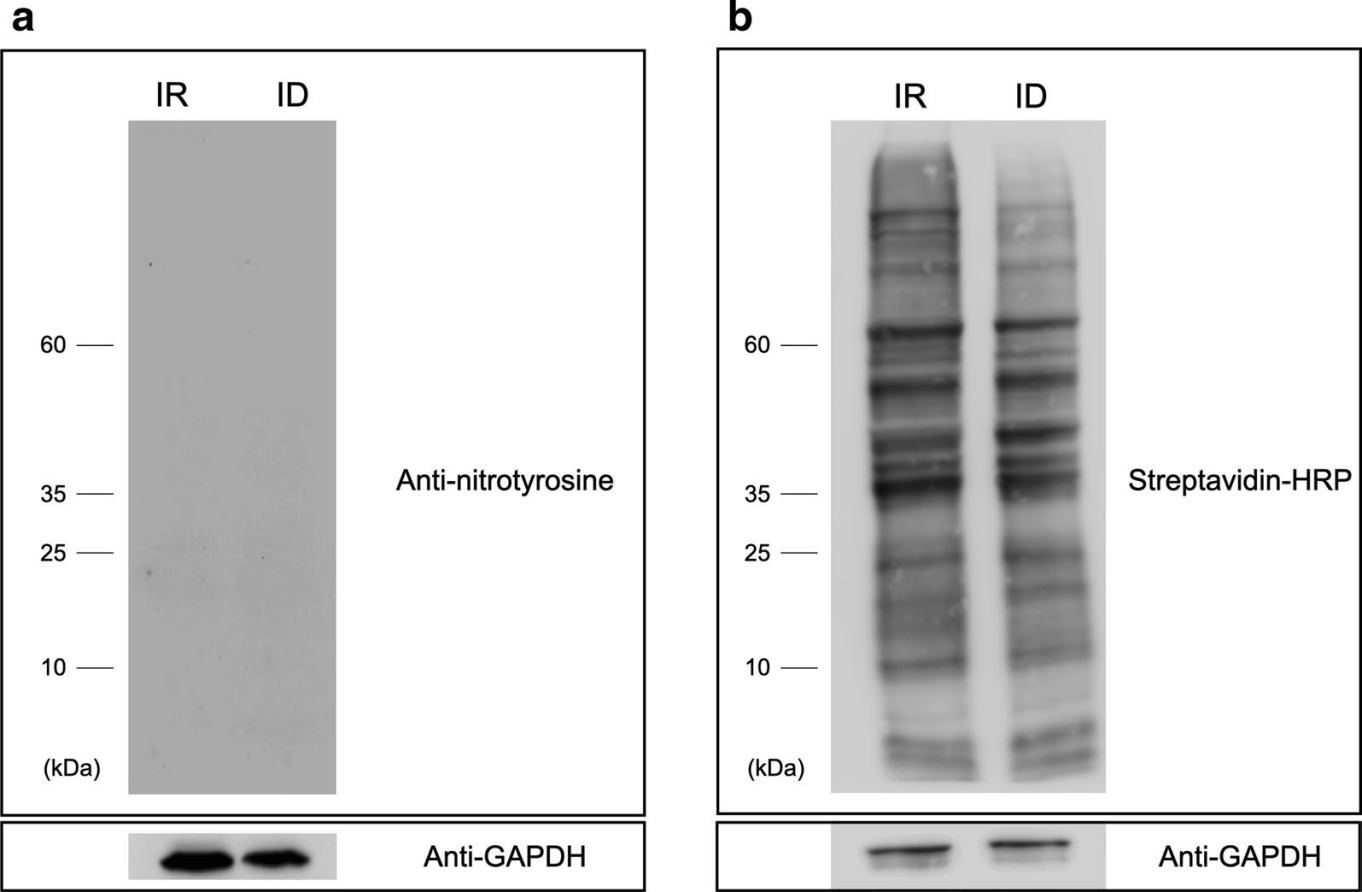
Protein cysteine S-nitrosylation and not tyrosine nitration occurs in *T. vaginalis*. **a** Detection of protein tyrosine nitration was performed by anti-nitrotyrosine western blotting. Proteins collected from iron-rich (IR) and -deficient (ID) cultured *T. vaginalis* were loaded (10 µg) and hybridized by anti-nitrotyrosine antibody (1:500). **b** Biotin-switch assay for measuring protein cystine S-nitrosylation of trichomonad proteins. The biotin-replaced proteins were visualized by streptavidin-HRP (1:5000). GAPDH was used as the loading control

We previously demonstrated that NO plays pivotal roles in *T. vaginalis* under ID conditions, promoting cell viability by maintaining hydrogenosomal quality and quantity [16]. NO and its derivatives modify proteins by tyrosine nitration and cysteine S-nitrosylation to initiate proper responses [29]. However, NO-dependent signaling in *T. vaginalis* has not been elucidated. We showed that there was no signal recognized by an anti-nitrotyrosine antibody, indicating that tyrosine nitration on proteins was absent in *T. vaginalis* (Fig. 3a). The biotin switch assay was performed to replace the S-nitrosothiol group (SNO) on trichomonad proteins to monitor the event of protein nitrosylation [19], and the biotin-containing proteins were detected by streptavidin HRP (Fig. 3b). The results showed that both IR and ID proteomes were modified with SNO despite the fact that the intensity of nitrosylated proteins was inconsistent with NO level [16]. Together, the results indicated that NO was capable of modifying the trichomonad proteomes through cysteine S-nitrosylation.

SNO proteome profiling of *T. vaginalis* cultured under different iron availabilities was conducted using LC-MS analysis. Seventy proteins were identified as S-nitrosylated in IR (61) and ID (45) trichomonad cells (Table 1). Most of the nitrosylated proteins were shared between the two conditions, with only few being unique to the IR or ID group. Among these identified proteins, ribosomal proteins (47%) and glycolytic enzymes (17%) were the major targets of SNO modification in *T. vaginalis*. This result indicated that trichomonad glycolytic enzymes were also modified by protein nitrosylation. As TvLDH was one of the nitrosylated proteins in ID cells, we sought to determine whether protein nitrosylation affects the function of TvLDH under ID conditions.

**Table 1:** Proteomics identification of cysteine S-nitrosylated proteins in *T. vaginalis*

Functional classification	Gene product	Gene ID	IR	ID
Protein synthesis/ribosomal proteins	Ribosomal protein L14	TVAG_026460	+	
	Ribosomal protein S13p/S18e	TVAG_020480	+	
	Putative translation initiation inhibitor	TVAG_035410	+	
	Ribosomal protein L24	TVAG_038050	+	
	Ribosomal protein S3	TVAG_106800	+	
	Ribosomal protein	TVAG_128790	+	
	40S ribosomal protein S4	TVAG_131210	+	
	RNA-binding protein	TVAG_158990	+	
	40S ribosomal protein S5	TVAG_163180	+	
	Ribosomal protein L13e	TVAG_423320	+	
	Ribosomal protein L8	TVAG_005910	+	+
	Ribosomal protein L29	TVAG_013870	+	+
	Ribosomal protein L36e	TVAG_020530	+	+
	40S ribosomal protein S6	TVAG_033590	+	+
	60S ribosomal protein L7-2	TVAG_054130	+	+
	Elongation factor 1-alpha	TVAG_067400	+	+
	60S ribosomal protein L30	TVAG_072050	+	+
	40S ribosomal protein S8	TVAG_066030	+	+
	Ribosomal protein L10a	TVAG_074480	+	+
	Ribosomal protein L22	TVAG_083260	+	+
	Ribosomal protein L5	TVAG_113720	+	+
	40S ribosomal protein S7	TVAG_143030	+	+
	Ribosomal protein L23	TVAG_160160	+	+
	Ribosomal protein L35Ae	TVAG_185880	+	+
	Ribosomal protein L7Ae	TVAG_199270	+	+
	Ribosomal protein S24e	TVAG_272960	+	+
	60S ribosomal protein L18a	TVAG_347250	+	+
	Ribosomal protein L34e	TVAG_417810	+	+
	Ribosomal protein S14	TVAG_464120	+	+
	Ribosomal protein L24e	TVAG_101690		+
	Ribosomal protein L13e	TVAG_112230		+
	Ribosomal protein L38e	TVAG_246730		+
	Ribosomal protein S19e	TVAG_352520		+
Carbohydrate metabolism	Pyruvate:ferredoxin oxidoreductase A-like protein (Fragment)	TVAG_198110	+	
	Malate dehydrogenase	TVAG_204360	+	
	Malate dehydrogenase	TVAG_253650	+	
	Phosphoglycerate kinase	TVAG_268050	+	
	Fructose-1,6-bisphosphate aldolase	TVAG_300000	+	
	Glyceraldehyde-3-phosphate dehydrogenase	TVAG_146910	+	+
	Hydrogenosomal malic enzyme subunit B proprotein	TVAG_238830	+	+
	Succinate-CoA ligase [ADP-forming] subunit alpha	TVAG_318670	+	+
	Enolase 2	TVAG_329460	+	+
	l-lactate dehydrogenase	TVAG_171090		+
	Phosphoenol pyruvate carboxykinase	TVAG_310250		+
	Fructose-1,6-bisphosphate aldolase	TVAG_345360		+
Cytoskeleton	Putative actin depolymerizing factor	TVAG_192620	+	
	Actin (Fragment)	TVAG_512800	+	+
Antioxidant	Thioredoxin reductase	TVAG_474980		+
Proteolysis	Ubiquitin	TVAG_069570	+	
Other	Adenosylhomocysteinase	TVAG_210320	+	
	QXW lectin repeat family protein	TVAG_261950	+	
	Cytosolic repetitive antigen	TVAG_427040	+	
	Plectin/S10 domain containing protein	TVAG_329340	+	+
	TolA	TVAG_411090	+	+
	HMG box family protein	TVAG_325010		+
Unknown	Uncharacterized protein	TVAG_196630	+	
	Uncharacterized protein	TVAG_198100	+	
	Uncharacterized protein	TVAG_210380	+	
	Uncharacterized protein	TVAG_219770	+	
	Uncharacterized protein	TVAG_539120	+	
	Uncharacterized protein	TVAG_010560	+	+
	Uncharacterized protein	TVAG_071700	+	+
	Uncharacterized protein	TVAG_083700	+	+
	Uncharacterized protein	TVAG_111510	+	+
	Uncharacterized protein	TVAG_121550	+	+
	Uncharacterized protein	TVAG_210010	+	+
	Uncharacterized protein	TVAG_226630	+	+
	Uncharacterized protein	TVAG_296920	+	+
	Uncharacterized protein	TVAG_306370	+	+
	Uncharacterized protein	TVAG_487100	+	+

*Notes*: SNO modified proteins identified in iron-rich (IR) and -deficient (ID) conditions by proteomics analysis were listed in this table. “+” represented the proteins `detected in IR and/ or ID conditions

### NO-dependent protein modification controlled the activity of glycolytic enzymes

Although we were unable to directly manipulate the NO level in *T. vaginalis*, we were able to increase the level of SNO-modified proteins by adding sodium nitrate [16, 30]. The staining intensity of SNO-modified proteins was enhanced after nitrate treatment (Additional file 5: Figure S2). Moreover, the viability of ID cells was elevated by 10% in the nitrate-treated groups after 24 h of incubation, suggesting that nitrate treatment was not toxic to ID cells (Additional file 5: Figure S2). These data confirmed that nitrate treatment was a suitable model for our examinations. The intensity of the SNO signal was not increased at a higher concentration of sodium nitrate (50 mM). Therefore, we utilized 25 mM sodium nitrate in the subsequent experiments.

**Fig. 4:**
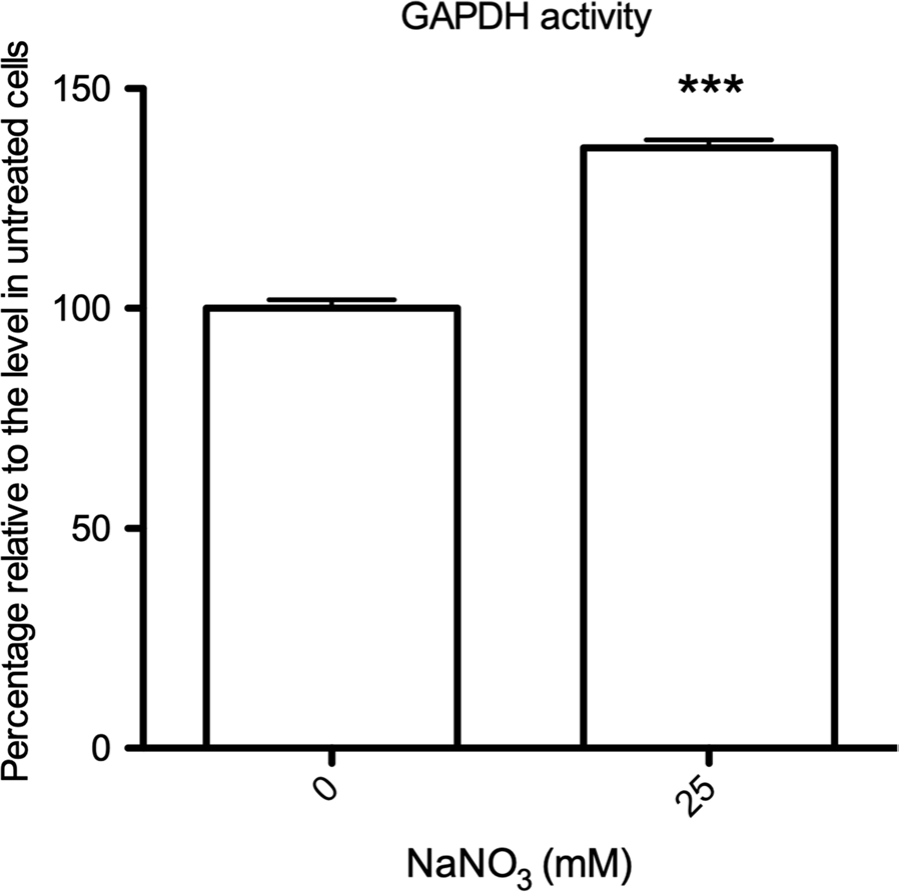
The activity of TvGAPDH was enhanced by sodium nitrate treatment in iron deficient (ID) *T. vaginalis*. TvGAPDH activity in ID cells was measured after sodium nitrate (25 mM) treatment for 6 h. The activity of TvGAPDH of the experimental group relative to that of untreated ID cells is shown

Higher levels of glycolysis in ID cells than in IR cells were observed, as evidenced by lactate accumulation. However, it remained unclear whether the activities of glycolytic enzymes were modified by protein nitrosylation. We assessed the activity of GAPDH, a SNO-modified glycolytic enzyme identified in both IR and ID conditions, after nitrate treatment (Table 1). The results showed that GAPDH activity was higher in the nitrate-treated ID cells than in the untreated ID cells (Fig. 4), indicating that the enzymatic activity was enhanced by SNO modification. We concluded that SNO modification enhanced the activity of glycolytic enzymes.

### TvLDH activity was enhanced by protein nitrosylation in *T. vaginalis*

**Fig. 5:**
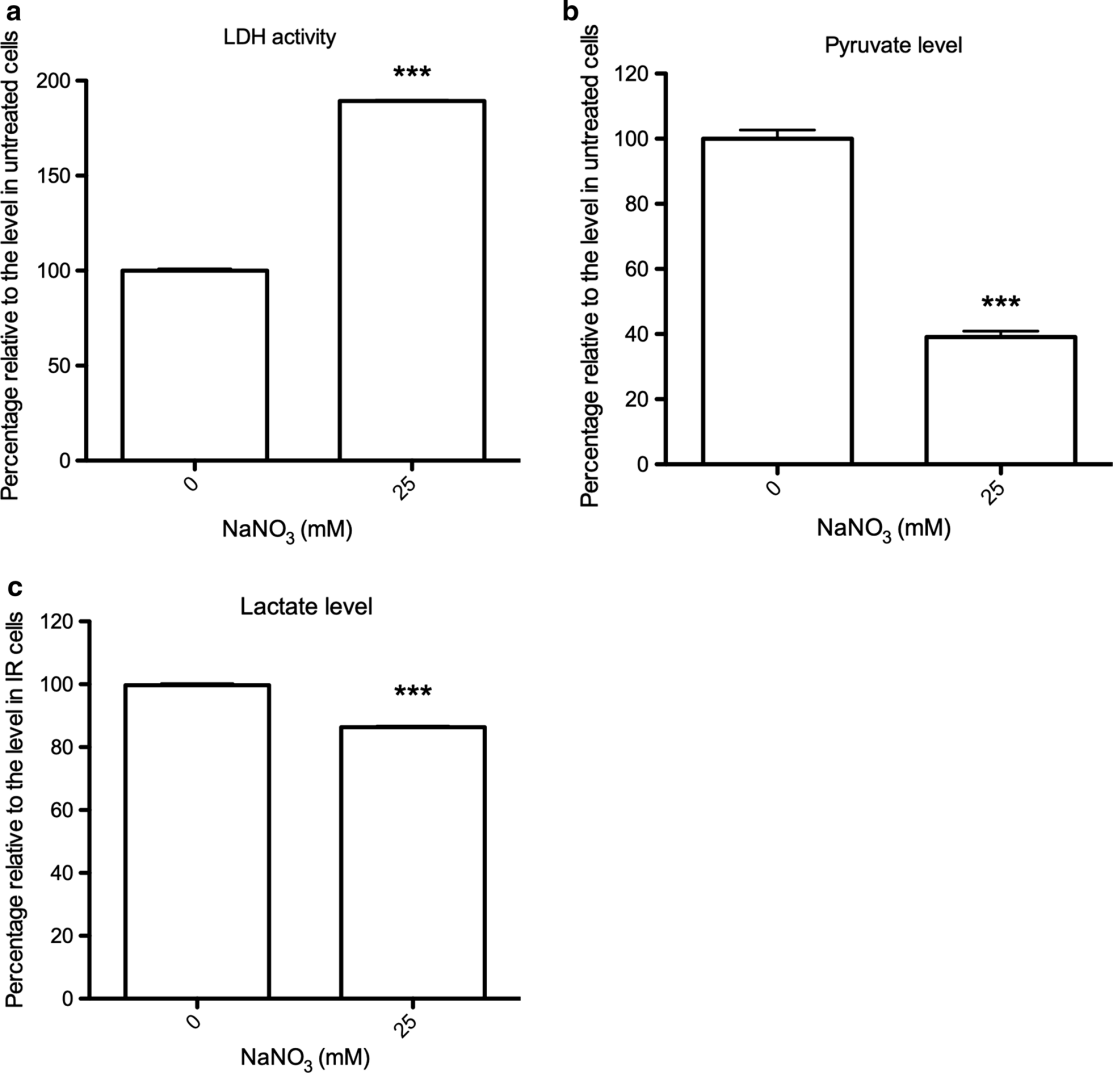
The activity of TvLDH was upregulated by sodium nitrate, and the catalytic direction was preferentially toward lactate formation. **a** The activity of TvLDH of iron-deficient (ID) cells treated with sodium nitrate (25 mM) for 6 h was measured. The activity of TvLDH in the experimental cells relative to that in untreated control cells is shown. **b** Pyruvate level in ID cells was measured after sodium nitrate (25 mM) treatment for 6 h. The amount of pyruvate in nitrate-treated ID cells relative to that in untreated control cells is shown. **c** Lactate level in ID cells was measured after sodium nitrate (25 mM) treatment for 6 h. The amount of lactate in nitrate-treated ID cells relative to that in untreated control cells is shown. *** *P* < 0.001 compared with the untreated control group (0 mM)

The extensive upregulation of TvLDH in ID trichomonads was considered responsible for catalyzing the conversion of pyruvate into lactate. We investigated whether protein nitrosylation affected the activity of TvLDH since it was an exclusive target of SNO modification in the ID treatment (Table 1). The activity of TvLDH was 2-fold higher in nitrate-treated cells than in untreated ID cells (Fig. 5a). To determine the catalytic direction of activated TvLDH, the levels of pyruvate and lactate were investigated. The pyruvate level showed a 60% decrement after nitrate treatment relative to control treatment in ID cells (Fig. 5b), suggesting that the catalytic direction of TvLDH toward lactate was due to pyruvate reduction. However, the lactate level was downregulated by ~ 15% in ID cells treated with nitrate relative to untreated cells (Fig. 5c). These results revealed that the significant elevation of TvLDH activity under iron deficiency was dependent on protein nitrosylation and that TvLDH preferentially catalyzed pyruvate reduction.

### The NAD^+^ to NADH ratio was regulated by protein nitrosylation in *T. vaginalis* under iron deficiency

**Fig. 6:**
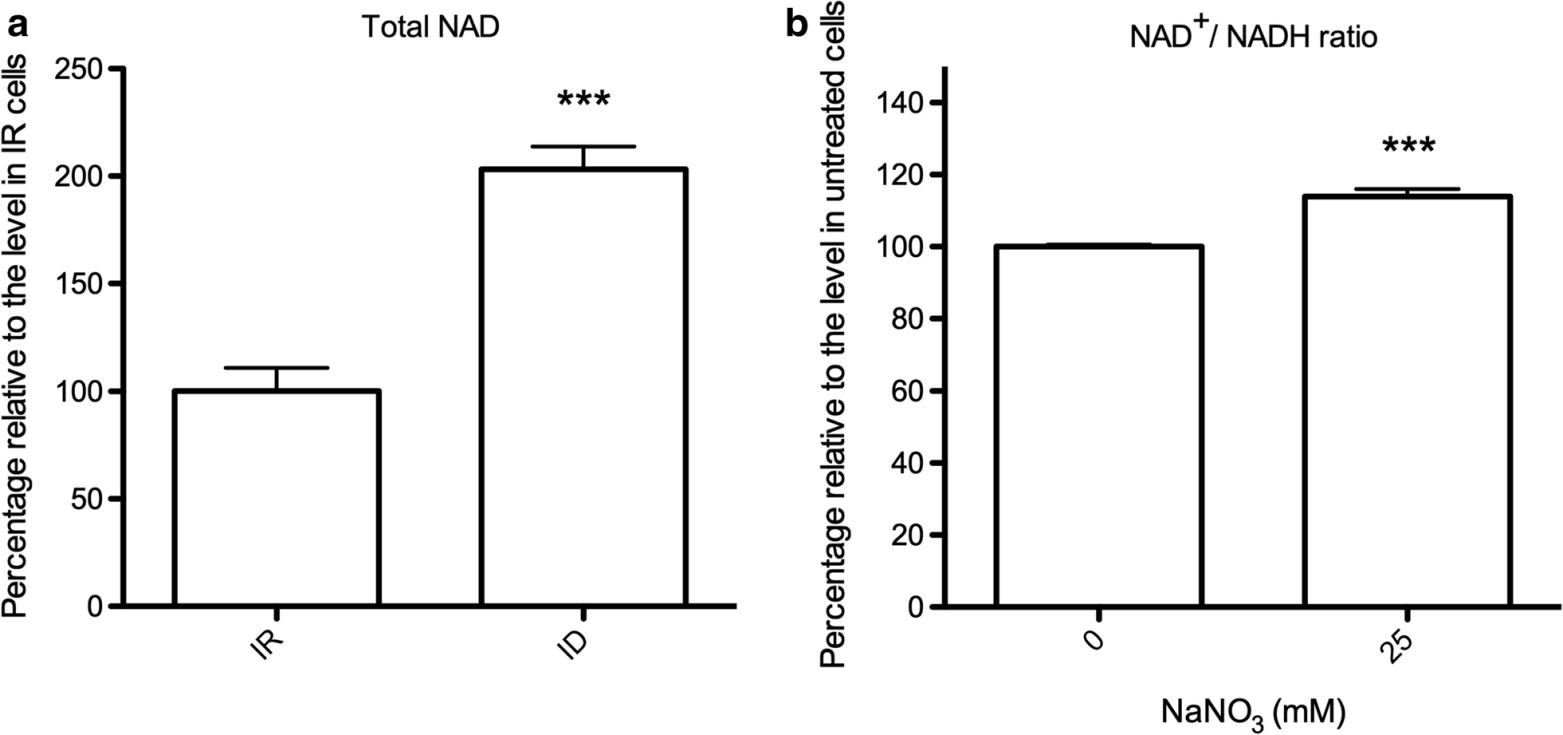
The NAD^+^ to NADH ratio was regulated by protein nitrosylation. **a** Total NAD levels in iron-rich control (IR) and iron-deficient (ID) groups were measured; the amount of total NAD in ID cells relative to IR cells is shown. *** *P* < 0.001 compared with the IR group. **b** The NAD^+^ to NADH ratio of ID cells treated with sodium nitrate (25 mM) was determined. The NAD^+^ to NADH ratio of ID cells treated with sodium nitrate relative to that of untreated control cells (0 mM) is shown. *** *P* < 0.001 compared with the untreated control group

LDH reduces pyruvate to lactate, and this reduction is accompanied by NAD^+^ oxidation, which provides additional reducing power for cells in iron-limited conditions [30]. Moreover, the direction catalyzed by TvLDH after SNO modification had yet been determined. We therefore assessed the amount of oxidized NAD^+^ to investigate NAD oxidoreduction status and the direction of NO-activated TvLDH. We measured the total amount of NAD and identified 2-fold increase in ID cells compared with IR control cells (Fig. 6a).

The amount of NAD was significantly accumulated upon iron deficiency; however, the NAD^+^ to NADH ratio is the measure that conveys functional information. To address this, we calculated the ratio of oxidized NAD to reduced NAD and found that NAD^+^ oxidation was positively correlated with nitrate treatment (Fig. 6b). This result suggested that the level of NAD, especially the oxidized form, was elevated in the presence of NO in *T. vaginalis* under iron-deficient conditions. In other words, protein nitrosylation was responsible for the oxidation of NAD^+^, which was achieved by enhancing TvLDH activity.

## Discussion

Glycolysis is a compensatory energy production and NAD recycling mechanism in mammals under iron shortage [10]. By monitoring lactate concentration, we demonstrated that iron deficiency enhances glycolysis in *T. vaginalis*. However, the expression of glycolytic enzymes did not appear to change under iron deficiency, leading us to assume that PTM plays a role in regulating the efficiency of glycolysis. Tyrosine nitration and cysteine S-nitrosylation are two NO-related PTMs. Similar to protein phosphorylation, these PTMs are expected to alter protein conformation, changing the molecular behavior of target proteins. We ruled out the possible occurrence of tyrosine nitration in *T. vaginalis*. Nitrotyrosine is mediated by peroxynitrite anion (ONOO^−^) and nitrogen dioxide (NO_2_), and these secondary compounds of NO are generated by incorporation with reactive oxygen species (ROS) [31]. This observation is in agreement with our previous observation that ROS level was not affected by iron concentration [16]. However, we found that SNO-modified proteins exist in *T. vaginalis*. The signal of the SNO proteome was more intense in the IR group than in the ID group, which was not consistent with observed NO levels. A similar phenomenon has been observed in epithelial NOS knockout mice, suggesting that other sources of NO contribute to protein nitrosylation [22]. Protein nitrosylation is a type of NO storage [32–34]. Thus, the presence of nitrosylated proteins might allow rapid responses to environmental changes, especially under iron shortage. This possibility warrants investigation in taxa with unknown NO-producing machinery.

The mechanism of protein nitrosylation in *T. vaginalis* has not been described. A previous report demonstrated that hybrid cluster protein (HCP) acts as a transnitrosylase to mediate protein SNO modification [35]. TvHCP (TVAG_336320) might be responsible for S-nitrosothiol transfer of proteins since it is strongly upregulated by iron deficiency [16]. Protein nitrosylation is reversible through thioredoxin (Trx) activity. Trx together with thioredoxin reductase (TrxR) participate in the denitrosylase system to remove nitrosothiol groups from SNO-modified proteins [36]. In our present study, SNO-modified TrxR was exclusively detected in ID cells. TrxR activity was inhibited by SNO modification, implying that the nitrosothiol groups could be retained under ID conditions [37].

More than ~ 50% of the SNO proteome comprised ribosomal proteins, consistent with previous studies [18, 19, 38]. Ribosomal proteins are considered to function in protein translation, but they can also act as nucleases, transcription factors, and mediators of cell signaling [39]. Glycolytic enzymes represented the second largest proportion of the SNO proteome, similar to the findings of most previous reports [18, 19, 38]. We demonstrated that the activities of TvGAPDH and TvLDH were enhanced after nitrate exposure, suggesting that protein nitrosylation plays a crucial role in the regulation of glycolytic enzymes.

Previous reports showed that the expression of TvLDH in *T. vaginalis* was greatly increased at both the mRNA and protein levels upon iron deficiency, implying a crucial function of TvLDH or lactate in iron-limited conditions [16, 40, 41]. TvLDH activity is induced by iron depletion; in addition, LDH has been shown to be nitrosylated in a mouse model [20, 22, 42]. These observations imply that both expression level and SNO modification regulate the function of TvLDH. We showed that the activity of TvLDH was regulated by NO signaling upon iron deficiency. The reduction of pyruvate also confirmed the enhanced active of TvLDH, although lactate level was also reduced by the same treatment. The accumulation of lactate impairs the growth and survival of eukaryotic cells since it causes lactic acidosis and eventually apoptosis [43]. Therefore, the bidirectional feature of TvLDH might be responsible for maintaining the dynamics of lactate to prevent cells from metabolic acidosis. Recently, accumulating evidence has revealed that the reverse direction of lactate to pyruvate enters the TCA cycle to contribute energy for cardiac muscle and brain [44, 45]. However, a beneficial effect of lactate on cell growth or viability in *T. vaginalis* was not observed in ID cells treated with excess lactate (data not shown).

Pyruvate is the central metabolic intermediate related to energy production, amino acid conversion, fatty acid biosynthesis, and gluconeogenesis. According to our previous transcriptomics analysis, alanine aminotransferase (TvALT) and alcohol dehydrogenase (TvADH) were upregulated in ID conditions, implying that the above reactions probably consume pyruvate [6, 16]. Pyruvate:ferredoxin oxidoreductase (PFO) was almost absent in trichomonad cells upon iron limitation, indicating that energy and acetyl-CoA-related metabolites, including fatty acids, are all reduced [7]. Gluconeogenesis is another metabolic direction of pyruvate. However, the key bypass enzymes for unidirectional steps of glycolysis, such as glucose-6-phosphatase and fructose-1,6-biphosphatase, are absent from the genome of *T. vaginalis* [46]. Moreover, the expression of pyruvate phosphate dikinase and phosphoenolpyruvate carboxykinase is downregulated upon iron shortage. These observations are concordant with the observed decrease in pyruvate carboxylase expression under iron depletion [47]. Hence, we excluded gluconeogenesis as a possibility in ID-treated trichomonad cells [16].

The reduction of pyruvate to lactate is accompanied by the oxidation of NAD, which provides the reducing power necessary for maintaining metabolic redox status [10, 30]. The enhancement of TvGAPDH and TvLDH activity following NAD oxidation has been demonstrated previously, indicating that intracellular NAD^+^ recycling is regulated by NO signaling. NAD^+^ is the coenzyme of the silent information regulator protein Sir2. Sir2 is a protein deacetylase that regulates a variety of biological functions, such as gene expression, chromatin management, and cell proliferation [12, 48]. There are 11 Sir2 homologs annotated in *T. vaginalis*, the expression of which is mostly upregulated by iron deficiency [16]. Mammalian Sir2 is capable of regulating iron homeostasis through deacetylating transcription factors and inhibiting the export of iron from cells [49]. PARP is another multifunctional protein that requires NAD^+^ as a cofactor for proper functionality. PARP mediates protein poly ADP-ribosylation, which is involved in DNA repair, the maintenance of chromatin structure, and apoptosis [13, 50]. The putative functions of Sir2 and PARP under increased NAD^+^ likely involve adaptation to environmental changes in iron levels. However, the mechanisms require further investigation.

## Conclusions

We demonstrated that iron deficiency accelerated the breakdown of glucose in *T. vaginalis*. By conducting cysteine S-nitrosylation proteomics analysis, we found that glycolytic enzymes were modified by SNO. The activities of TvGAPDH and TvLDH were enhanced by nitrate addition. Furthermore, nitrosylated TvLDH preferentially catalyzed pyruvate reduction and NAD^+^ regeneration. Our findings suggest that in *T. vaginalis* under iron shortage, protein nitrosylation is crucial for providing the reducing power necessary to sustain biological functions and to compensate for redox imbalance *via* protein nitrosylation of TvLDH.

## Supplementary information

**Additional file 1: Table S1.** Primer sets used for quantitative real-time PCR.

**Additional file 2: Table S2.** The expression patterns of glycolytic enzymes in *T. vaginalis* cultured in iron-rich (IR) and -deficient (ID) conditions. The expression changes of all glycolytic enzymes in iron-deficient (ID) conditions relative to their expression in iron-rich control (IR) cells. The log 2-fold-changes (ID/IR) are shown; the background color indicates the upregulation (red) or downregulation (green) of each gene.

**Additional file 3: Alignment S1.** Sequence alignment of TvLDHs and TvMDHs. All of the TvLDHs and TvMDHs were aligned as shown. The protein sequences containing leucine 91 (L91) are highlighted in red, and the accession numbers are presented in bold.

**Additional file 4: Figure S1.** The mapping results of our previous next-generation RNA sequencing of TVAG_171090 and TVAG_171100. The short reads generated from the sequencing were mapped to the reference sequences (top of the figure, black), and the locations of individual reads are shown. Blue and light blue, unique paired-ended reads; green, forward single-ended reads; red, reverse single-ended reads; yellow, non-specific reads. Only the first 300 rows are shown in this figure.

**Additional file 5: Figure S2.** The effects of sodium nitrate treatment on ID *T. vaginalis*. **a** The SNO proteomes of ID *T. vaginalis* treated with 25 and 50 mM of sodium nitrate and incubated for 6 h. **b** The viability of sodium nitrate-treated *T. vaginalis* after a 24 h incubation.

## Data Availability

All data generated or analyzed during this study are included in the published article and its additional files.

## Abbreviations

NO: nitric oxide
ID: iron deficiency
IR: iron rich
LDH: lactate dehydrogenase
PCR: polymerase chain reaction
SNO: S-nitrosothiol
GAPDH: glyceraldehyde-3-phosphate dehydrogenase
NAD^+^: oxidized nicotinamide adenine dinucleotide
NADH: reduced nicotinamide adenine dinucleotide
PTM: post-translational modification
LC-MS: liquid chromatography mass spectrometry
STI: sexually transmitted infection
HIV: human immunodeficiency virus
PFO: pyruvate: ferredoxin oxidoreductase
acetyl-CoA: acetyl coenzyme A
TCA cycle: tricarboxylic acid cycle
PARP: poly (adenine dinucleotide phosphate-ribose) polymerase
NOS: nitric oxide synthase
DIP: dipyridyl
YI-S medium: yeast extract, iron-serum medium
FAC: ferrous ammonium citrate
MDH: malate dehydrogenase
DEPC: diethylpyrocarbonate
cDNA: complementary DNA
ELISA: enzyme-linked immunosorbent assay
TBS: tris-buffered saline
HRP: horseradish peroxidase
SDS-PAGE: sodium dodecyl sulfate polyacrylamide gel electrophoresis
TTBS: Tris-buffered saline Tween-20
ECL: enhanced chemiluminescence
ABC: ammonium bicarbonate
DTT: dithiothreitol
IAM: iodoacetamide
HPLC: high performance liquid chromatography
CID: Collision-induced dissociation
PSM: peptide-spectrum match
PBS: phosphate buffered saline
RNA-seq: RNA sequencing
ROS: reactive oxygen species
HCP: hybrid cluster protein
Trx: thioredoxin
TrxR: thioredoxin reductase
ALT: alanine aminotransferase
ADH: alcohol dehydrogenase
Sir2: sirtuin, silent information regulator protein
