# Supplementary material for: Protein cysteine S-nitrosylation provides reducing power by enhancing lactate dehydrogenase activity in Trichomonas vaginalis under iron deficiency
Source: Parasit Vectors. 2020 Sep 18;13:477. doi: 10.1186/s13071-020-04355-0 (PMC7501694; doi:10.1186/s13071-020-04355-0)
Supplement: Supplementary file 1 — Additional file 1: Table S1. Primer sets used for quantitative real-time PCR. [file 13071_2020_4355_MOESM1_ESM.pdf]

**Additional file 1: Table S1.** Primer sets used for quantitative real-time PCR.

| Gene ID | Forward (5'-3') | Reverse (5'-3') |
|---|---|---|
| TVAG_534540 | TCGGAACAAAGCCAGGAGAA | ACTCTGCGAGGTGGTTCAAA |
| TVAG_171090 | TCAAGTTCGTGCAGACCT | TCGAGGAGTGAGAGGCTAGA |
| TVAG_455680 | GATAGCAGGAGGCGAGCTTT | GCTGATGAGGTCAGCACGAA |
| TVAG_239990 | TCCCATTGGATTGCAGGAGG | AGTTGCGATGTAGCCAGCAA |
| TVAG_371320 | AGGTCACCAATTGAGAGGTCGT | GTAACCTGCGAGGTGTGGGA |
| TVAG_381310 | TGGCTTTTCGGAACCAAGGA | CTCCTTGAGCCAGTCGTTGA |
| TVAG_495880 | CTTCGGAACAGCCCCAGGAG | CGCGCTTATTGAGCAAGGTGG |
