# Supplementary material for: Protein cysteine S-nitrosylation provides reducing power by enhancing lactate dehydrogenase activity in Trichomonas vaginalis under iron deficiency
Source: Parasit Vectors. 2020 Sep 18;13:477. doi: 10.1186/s13071-020-04355-0 (PMC7501694; doi:10.1186/s13071-020-04355-0)
Supplement: Supplementary file 2 — Additional file 2: Table S2. The expression patterns of glycolytic enzymes in T. vaginalis cultured in iron-rich (IR) and -deficient (ID) conditions. The expression changes of all glycolytic enzymes in iron-deficient (ID) conditions relative to their expression in iron-rich control (IR) cells. The log 2-fold-changes (ID/IR) are shown; the background color indicates the upregulation (red) or downregulation (green) of each gene. [file 13071_2020_4355_MOESM2_ESM.pdf]

Additional file 2: Table S2. The expression patterns of glycolytic enzymes in *T. vaginalis* cultured in iron-rich (IR) and -deficient (ID) conditions. The expression changes of all glycolytic enzymes in iron-deficient (ID) conditions relative to their expression in iron-rich control (IR) cells. The log 2-fold-changes (ID/IR) are shown; the background color indicates the upregulation (red) or downregulation (green) of each gene.

Log2 fold-change (ID/IR)

Glucokinase	
TVAG_092750	0.43
TVAG_397250	-0.11
TVAG_258370	-0.67
TVAG_336940	3.33
TVAG_282870	0.14
TVAG_204370	-1.58
TVAG_045010	0.57
TVAG_260790	-0.78
TVAG_188820	-2.94

Glucose-6-phosphate isomerase	
TVAG_061930	1.24
TVAG_389760	0.53

Phosphofructokinase	
TVAG_430830	-1.32
TVAG_079260	-0.77
TVAG_364620	0.44
TVAG_462920	0.12
TVAG_391760	-0.59
TVAG_496160	0.14
TVAG_293770	1.36
TVAG_281070	-1.75
TVAG_263690	0.50
TVAG_077440	-1.27
TVAG_335880	0.61

Triosephosphate isomerase	
TVAG_096350	0.56
TVAG_497370	0.54

GAPDH	
TVAG_193800	1.37
TVAG_146910	0.34
TVAG_412780	-0.71
TVAG_366380	2.33
TVAG_475220	0.73
TVAG_347410	0.38
TVAG_476100	0.89
TVAG_193790	-0.30

Phosphoglycerate kinase	
TVAG_383940	-0.37
TVAG_268050	-0.15

Phosphoglycerate mutase	
TVAG_113710	-0.41
TVAG_212740	-1.79
TVAG_209020	-1.75
TVAG_120730	0.33
TVAG_165570	-0.81

Enolase	
TVAG_464170	0.18
TVAG_043500	0.99
TVAG_263740	-0.38
TVAG_358110	-2.36
TVAG_487600	-0.76
TVAG_148010	-0.36
TVAG_170370	-0.17
TVAG_282090	-1.44
TVAG_329460	6.15

Pyruvate kinase	
TVAG_373720	1.04
TVAG_015950	-0.60

6
0
-6
