# Supplementary material for: Protein cysteine S-nitrosylation provides reducing power by enhancing lactate dehydrogenase activity in Trichomonas vaginalis under iron deficiency
Source: Parasit Vectors. 2020 Sep 18;13:477. doi: 10.1186/s13071-020-04355-0 (PMC7501694; doi:10.1186/s13071-020-04355-0)
Supplement: Supplementary file 3 — Additional file 3: Alignment S1. Sequence alignment of TvLDHs and TvMDHs. All of the TvLDHs and TvMDHs were aligned as shown. The protein sequences containing leucine 91 (L91) are highlighted in red, and the accession numbers are presented in bold. [file 13071_2020_4355_MOESM3_ESM.pdf]

Additional file 3: Alignment S1. Sequence alignment of TvLDHs and TvMDHs. All of the TvLDHs and TvMDHs were aligned as shown. The protein sequences containing leucine 91 (L91) are highlighted in red, and the accession numbers are presented in bold.

```
TVAG_165030     ----MEKPA---HVL---------IVGAS-GQ----------IGYI-------LA-HWIAHGD------------------------LYG------------------DRQVFLHLYS--RTQDKLTALVME--LEDCAFPHLSGCVASTDPAVAF----
TVAG_171090     -MSEA---A---HVL---------ITGAA-GQ----------IGYI-------LS-HWIASGE------------------------LYG------------------ERKVYLHLFDIPPAINRLTALTME--LEDCAFPRLAGFVATTEPEQAF----
TVAG_171100     -MSEA---A---HVL---------ITGAA-GQ----------IGYI-------LS-HWIASGE------------------------LYG------------------ERKVYLHLFDIPPAINRLTALTME--LEDCAFPRLAGFVATTEPEQAF----
TVAG_193000     --MEP---V---HVL---------ITGAA-GQ----------IGYV-------LT-FRIAAGD------------------------LFG------------------DRKVVLHLLEIPQGMQALQGCCME--LQDCAFPAVAGIVATDKIEEAF----
TVAG_204360     --MEP---I---HIL---------ITGAA-GQ----------IGYA-------LT-FRIAKGD------------------------LCG------------------DRKVVLHLLEITPGMKALEGCVME--LQDCAFPNVAGIVWTDKVEEAF----
TVAG_239990     ----MSEPA---HVL---------ITGAA-GQ----------IGYI-------LS-HWIAGGE------------------------LYG------------------DRPVVLHLFDIPPAMNRLTALTME--LEDCAFPHLAGYIATTEPEAAF----
TVAG_253650     --MEP---I---HIL---------ITGAA-GQ----------IGYA-------LT-FRIAKGD------------------------LCG------------------DRKVVLHLLEIPFGMKALEGCVME--LQDCAFPNVAGIVWTDKVEEAF----
TVAG_354940     -MTES---V---RVL---------ISGAA-GQ----------IGYV-------MS-HWIADGM------------------------LFG------------------QRKIILHLFESTSAQSRLDALAME--LQDSSFRYLDGVVATTNPEEGF----
TVAG_362930     ----MVEPA---RVL---------ITGAA-GQ----------IGYV-------LS-HWIASGE------------------------LYG------------------ERPVILHLFDIPVAQNRLTALTME--LQDCAFPHLAGYVATTEPEQAF----
TVAG_371320     ----MSDPA---HVL---------ITGAA-GQ----------IGYI-------LS-HWIAGGE------------------------LYG------------------DRPVVLHLFDIPPAMNRLTALTME--LEDCAFPHLAGYVATTEPEAAF----
TVAG_381310     ----MTSPA---HVL---------ITGAA-GQ----------IGYV-------LS-NWIASGE------------------------LYG------------------NRPVVLHLFDIPVAENRLKALVME--LEDGAFQHLSGIVATTEPEKAF----
TVAG_387970     -MTQP---L---HVL---------VTGAA-GQ----------IGYV-------LA-FRIANGD------------------------LFG------------------ERDVVLHLLEISPAMKALEAVVME--LHDCTFPHLLHVIGTSDLEEAF----
TVAG_416100     MLTSV---SYPVRNICRSKLPLAKTGMTLLQDGDLSKGTAFTKEERDHLNLRGLLPYKVFTKDEQAARIRRQFELMPTPLLKYIFLANEREKNSQSFWRFLFTHPPEETMPVLYTPTVGEACQKW--ATHRQSYRGIYITPEDSG-----KIKDILHNYP
TVAG_455680     ----MSEPA---HVL---------ITGAA-GQ----------IGYI-------LS-HWIAGGE------------------------LYG------------------DRPVVLHLFDIPPAMNRLTALTME--LEDCAFPRLAGYVATTEPEAAF----
TVAG_495880     -MSEA---A---HVL---------ITGAA-GQ----------IGYI-------LS-HWIASGE------------------------LYG------------------DRQVYLHLLDIPPAMNRLTALTME--LEDCAFPHLAGFVATTDPKAAF----
TVAG_534540     -MSEA---A---HVL---------ITGAA-GQ----------IGYI-------LS-HWIASGE------------------------LYG------------------ERPVYLHLFDIPPAMNRLTALTME--LQDCAFPHLAGFVATTEPEAAF----
                            : :            :   :                     :    :                                                    :           .   .   :                  .    :

TVAG_165030     -KDVECAFLTSSYKL-PA---------------------------------------------------WEVRSFLVNKNSHIYKEVGEWLSMYANPSCKVLMIANPTNTNAALC--AKFAKNLNPQNFTSL---SMLDHNRANFY-IADKLG--VPVCD
TVAG_171090     -KDIDCAFLVASMPLKPG----------------------------------------------------QVRADLISSNSVIFKNTGEYLSKWAKPTVKVLVIGNPDNTNCEIA--MLHAKNLKPENFSSL---SLLDHNRA-YYEIASKLG--VSIND
TVAG_171100     -KDIDCAFLVASMPLKPG----------------------------------------------------QVRADLISSNSVIFKNTGEYLSKWAKPTVKVLVIGNPDNTNCEIA--MLHAKNLKPENFSSL---SLLDHNRA-YYEIASKLG--VSIND
TVAG_193000     -KDVDVAFLVGAFPRKDG----------------------------------------------------MDRADLLGKNGGIFTVQGKTLSDYAKPDVKVLVVGNPANTNALIA--LASAPKLGPKNFCAM---TRLDHNRM-LGELSVKLG--VPTNE
TVAG_204360     -KDVDVAFLVGSFPRKDG----------------------------------------------------MDRSDLLAKNGGIFTVQGKALNDYAKPTVKVLVVGNPANTNCLIA--QASAPKLQKKNWCAM---TRLDHNRM-VGALAAKFG--VTPEH
TVAG_239990     -KDIDCAFLVASMPLKPG----------------------------------------------------QVRADLISSNSVIFKNTGEYLSKWAKPTVKVLVIGNPDNTNCEIA--MLHAKNLKPENFSSL---SMLDHNRA-YYEVASKLG--VDIHD
TVAG_253650     -KGVDVAFLVGSFPRKDG----------------------------------------------------MDRSDLLAKNGGIFTVQGKALNDYAKPTVKVLVVGNPANTNCLIA--QASAPKLQNKNWCAM---TRLDHNRM-VGALAAKFG--VTPEK
TVAG_354940     -KDVDVAFLLCTCFTRPG----------------------------------------------------QKRLDILANNAKIYKQQGEYLNKYAKPTCKVLVTGNPANTNALVT--LLNAPNLKPENFTSL---SYLDHLRI-TNAVAIKLN--IESSK
TVAG_362930     -KDIDCAFLVASVPMKSG----------------------------------------------------QIRSDLIGSNSIIFKNTGEWLSQYAKPTVKVLVIGNPDNTNAEIA--LLHAKNLKPENFSSL---SLLDQNRA-YHAIAEKLG--VKVTD
TVAG_371320     -KDIDCAFLVASMPLKPG----------------------------------------------------QVRADLISSNSVIFKNTGEYLSKWAKPTVKVLVIGNPDNTNCEIA--MLHAKNLKPENFSFL---SMLDHNRA-YYEVASKLG--VHVHD
TVAG_381310     -KDIDCAFLVASMPLKPG----------------------------------------------------QVRSDLISSNSVIFKNTGEYLSQYAKPTVKVLVIGNPDNTNCEIA--MLHAKNLKPENFSSL---SMLDHNRA-YYEVAHKLG--VSIED
TVAG_387970     -RDVDVAFLVGSFPKKPS----------------------------------------------------TKLVDYFQRNASIYSEHGRALSDFAKPTVKVLVIGMPTNTNALVA--MTAAVNLSPKNFCAM---TRLDHNRA-VYSIAQKLG--VHHSK
TVAG_416100     RQDIRCIVVTDAGRILGLGDLGASGLGIPVGKLMLYTLIGQVNPDQTLPVQLDMGTDRKEILADPLYHGWRHPRIRGPAHTKFVEEFVAAVKEVFGETCLVQFEDFEMETAFTLLDHFRWR-----CNCFNDDIEGTAAVAAA-TLASATHMEGVPD-LK
TVAG_455680     -KDIECAFLVASMPLKPG----------------------------------------------------QVRADLISSNSVIFKNTGEYLSKWAKPTVKVLVIGNPDNTNCEIA--MLHAKNLKPENFSSL---SMLDHNRA-YYEVASKLG--VDIHD
TVAG_495880     -KDIDCAFLVASMPLKPG----------------------------------------------------QVRADLISSNSVIFKNTGEYLSKWAKPSVKVLVIGNPDNTNCEIA--MLHAKNLKPENFSSL---SMLDQNRA-YYEVASKLG--VDVKD
TVAG_534540     -KDIDCAFLVASMPLKPG----------------------------------------------------QVRADLISSNSVIFKNTGEYLSQWAKPTVKVLVIGNPDNTNCEIA--MLHAKNLTADNFSSL---SMLDHNRA-YYEVASKLG--VDIHD
                 :.:   .:  :                                                                   :  :       :.        * .     :*   :             *                    : ::       .

TVAG_165030     LKDVIVWGNHGETIVPDLTN-ATFVKN-GKTERILDVLGEDFMKNEFYKFMTSRGPCITKHRGISAAASTVVAVLEHMKALLFGTK--K-ILSLAIPVPENNKYGIKPGIVFSFPCTNDEDGKVNIVD-YPVNDWLQEKLKVTEKDLVDELKFALETLQE
TVAG_171090     IHDIIVWGNHGESMVSDLTQ-ATFTKD-GKTQKVVDVLDKEYVFDAFFKKIGHRVWDILEHRGFTSAASPTKAAIQHMKAWLFGTAPGE-VLSMGIPVPEGNPYGIKPGVVFSFPCSVDKEGHVHVVDGFKINDWLKEKLEFTEKDLFHEKEIALQHLAK
TVAG_171100     IHDIIVWGNHGESMVSDLTQ-ATFTKD-GKTQKVVDVLDKEYVFDAFFKKIGHRVWDILEHRGFTSAASPTKAAIQHMKAWLFGTAPGE-VLSMGIPVPEGNPYGIKPGVVFSFPCSVDKEGHVHVVDGFKINDWLKEKLEFTEEDLFHEKEIALQHLAK
TVAG_193000     IHKVTIWGNHSNTQVPDVSH-AVYGKE---NKKVSEALQAEYYQGEFVTKISTRGGAVIKMRGASSAASAANAALEHMRSWCFGT-PADDWVSMAIPVPENEPYGIKKGVIYSFPCTVDKEG-IHVVEGLAINDWVRGKMEATEKELVGEKETAWKVLGL
TVAG_204360     IHKVCVWGNHSNTQVPDTTH-ATVD-LPEGTVKVADKLPKEYLEGEFAQMIATRGGAVIKMRGASSAASAANAALTCVHDWLYGT-KEGDFVSMAIPVPDNEPYGIKQGIIFSFPVTVSKDGEVHVVEGLELNDCVKGKLEATEKELIGEKETAWKVLGL
TVAG_239990     VHDIIVWGNHGESMVADLTQ-ATFTKD-GKTQKVIDVLDHDYVFDTFFKKIGHRAWDILEHRGFTSAASPTKASIQHMKAWLFGTAPGE-VLSMGIPVPEDNPYGIKPGVVFSFPCNVDKEGKIHVVEGFKVNDWLREKLNFTENDLFHEKEIALQHLSE
TVAG_253650     IHKVCIWGNHSNTQVPDTTH-ATVD-LPEGTVKVADKLPKEYLEGEFAQMIATRGGAVIKMRGASSAASAANAALTCVKDWLYGT-AEGDFVSMAIPVPDNKPYGIKQGTIFSFPVTVSKDGEVHVVEGLELNDWVKGRLEATEKELIGEKETAWKVLGL
TVAG_354940     IRNVIVWGNHSDTLFPDISQ-AYFEKD-GEKNFVSSLLEEKYIHEELPDYISKRGWQILNYRGLSSASSPCLAAILTMKAFLFGTEPDQ-IISMGAIVPQSSPYGLQPGLLCSLPCTVDKDGKVHIVEDLQLNDWVNSKLKATEEELIQEKEYAMSII--
TVAG_362930     LHDIVVWGNHGESMVADLTQ-ATFEKD-GKIQKVTDVLDEKYREETFFKFISHRGWDIVEYRGFSSAASPTKASIQHMKAWLFGTKPGE-VLSMGIPVPENNKYGLKPGVVFSLPCTVDKDGNVHVVETYKVNDWLREKLEFTEKDLFNEKEIALHTLGE
TVAG_371320     VHDIIVWGNHGESMVADLTQ-ATFTKD-GKTQKVVDVLDHDYVFGTFFKKIGHRAWDILEHRGFTSAASPTKAAIQHMKAWLFGTAPGE-VLSMGIPVPEDNPYGIKPGVVFSFPCNVDKEGKIHVVDGFKVNDWLREKLDFTMNDLFHEKEIALEHLAQ
TVAG_381310     VHDIIVWGNHGESMVADLTN-ATFTKD-GKTQKVSDVLDEKYMYDTFFKFISHRAWDILEHRGFTSAASPTRAAIMHMKAWLFGTKENE-VLSLGIPVPEGNSYGIKPGVVFSFPCKVDKEGHIHVVDGYKINDWLKEKLAFTEDDLFKEKEIALRHLNE
TVAG_387970     VYKVVIWGNRSSSQIPDVSN-AEYQDE-TGRHHILEKVSEEYVNTEFSSDLAQRGNKVTMMRGASSAASAATAAIQCMRDWLYGT-PADNFVSMSVPVPESSPYGVKPGIFFSFPCTVDKEGNIHVVEGLPVDEATAKKIKQQEEEILNE----LKILAT
TVAG_416100     NQKIIFIGAGSAATGIANLIVDMAVSR-GGITKEQAYKNIIMFDHKGMVHAGR------------------------KD---------------------LYD---FNKPY----MHN----ME----V-Y-----------GSVLEGVKK---------
TVAG_455680     VHDIIVWGNHGESMVADLTQ-ATFTKD-GKTQKVVDVLDHDYVFDTFFKKIGHRAWDILEHRGFTSAASPTKASIQHMKAWLFGTAPGE-VLSMGIPVPEDNPYGIKPGVVFSFPCNVDKEGKIHVVEGFKVNDWLREKLNFTENDLFHEKEIALQHLGE
TVAG_495880     VHDIIVWGNHGESMVADLTQ-ATFTKE-GKTQKVVDVLDHDYVFDTFFKKIGHRAWDILEHRGFTSAASPTKAAIQHMKAWLFGTAPGE-VLSMGIPVPEGNPYGIKPGVVFSFPCNVDKEGKIHVVEGFKVNDWLREKLDFTEKDLFHEKEIALNHLAQ
TVAG_534540     VHDIIVWGNHGESMVADLTQ-ATFTKE-GKTQKVVDVLDKEYVFDTFFKKIGHRAWDILEHRGFTSAASPTKAAIQHMKAWLFGTKPGE-VLSMGIPVPEGNPYGIKPGVVFSFPCNVDKEGHIHVVDGFKVNDWLREKLDFTEKDLFHEKEIALNHLAE
                  .: . *  . :                                                                                                                                        :

TVAG_165030     Q-----
TVAG_171090     Q-----
TVAG_171100     Q-----
TVAG_193000     ------
TVAG_204360     ------
TVAG_239990     ------
TVAG_253650     ------
TVAG_354940     ------
TVAG_362930     IINLYH
TVAG_371320     ------
TVAG_381310     ------
TVAG_387970     PEE---
TVAG_416100     ------
TVAG_455680     ------
TVAG_495880     ------
TVAG_534540     ------
```
