# Supplementary material for: Protein cysteine S-nitrosylation provides reducing power by enhancing lactate dehydrogenase activity in Trichomonas vaginalis under iron deficiency
Source: Parasit Vectors. 2020 Sep 18;13:477. doi: 10.1186/s13071-020-04355-0 (PMC7501694; doi:10.1186/s13071-020-04355-0)

Additional file 4: Figure S1. The mapping results of our previous next-generation RNA sequencing of TVAG_171090 and TVAG_171100. The short reads generated from the sequencing were mapped to the reference sequences (top of the figure, black), and the locations of individual reads are shown. Blue and light blue, unique paired-ended reads; green, forward single-ended reads; red, reverse single-ended reads; yellow, non-specific reads. Only the first 300 rows are shown in this figure.

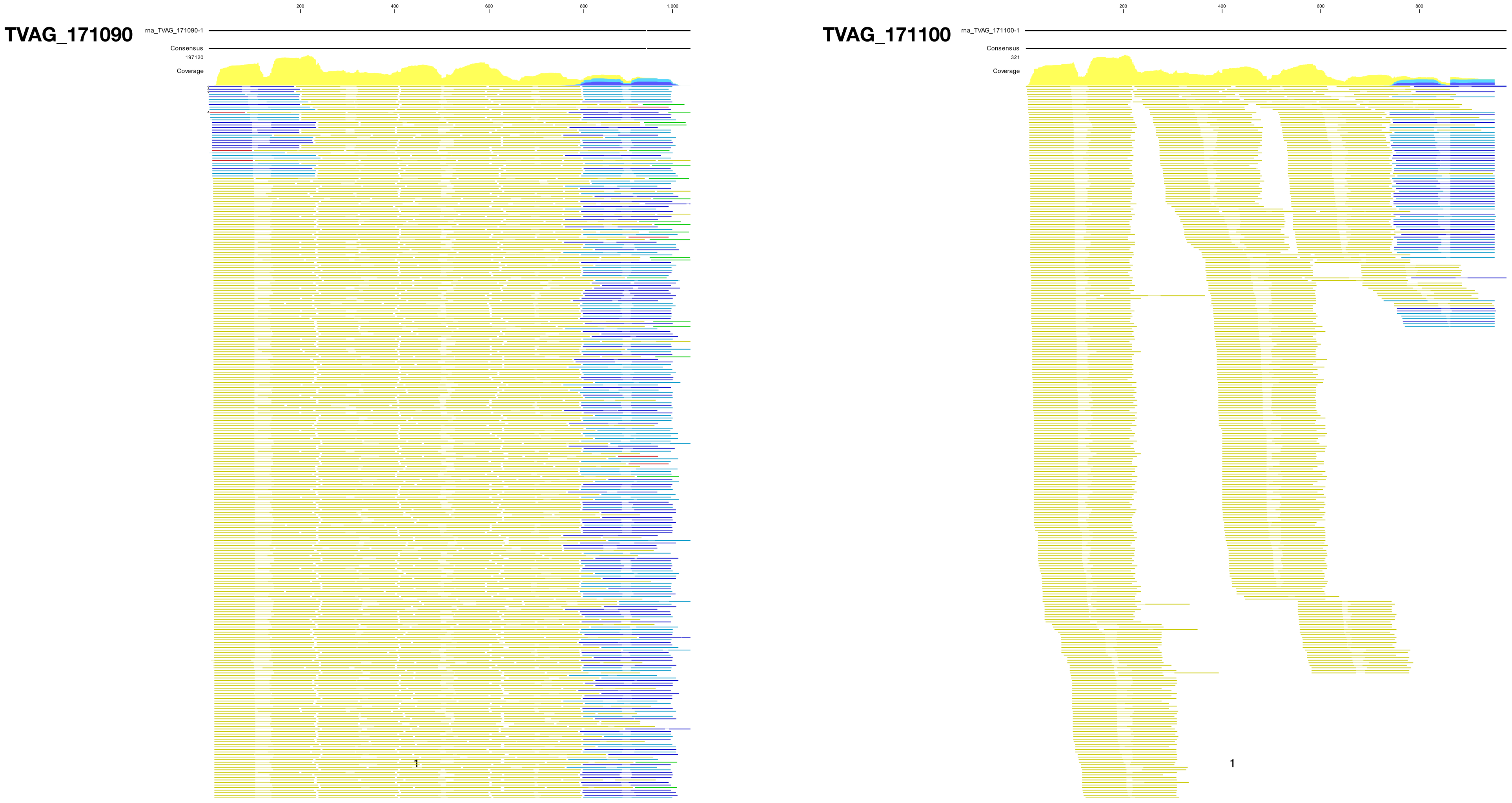

Supplement: Supplementary file 4 — Additional file 4: Figure S1. The mapping results of our previous next-generation RNA sequencing of TVAG_171090 and TVAG_171100. The short reads generated from the sequencing were mapped to the reference sequences (top of the figure, black), and the locations of individual reads are shown. Blue and light blue, unique paired-ended reads; green, forward single-ended reads; red, reverse single-ended reads; yellow, non-specific reads. Only the first 300 rows are shown in this figure. [file 13071_2020_4355_MOESM4_ESM.pdf]
