# Supplementary material for: Protein cysteine S-nitrosylation provides reducing power by enhancing lactate dehydrogenase activity in Trichomonas vaginalis under iron deficiency
Source: Parasit Vectors. 2020 Sep 18;13:477. doi: 10.1186/s13071-020-04355-0 (PMC7501694; doi:10.1186/s13071-020-04355-0)

Additional file 5: Figure S2. The effects of sodium nitrate treatment on ID *T. vaginalis*. **a** The SNO proteomes of ID *T. vaginalis* treated with 25 and 50 mM of sodium nitrate and incubated for 6 h. **b** The viability of sodium nitrate-treated *T. vaginalis* after a 24 h incubation.

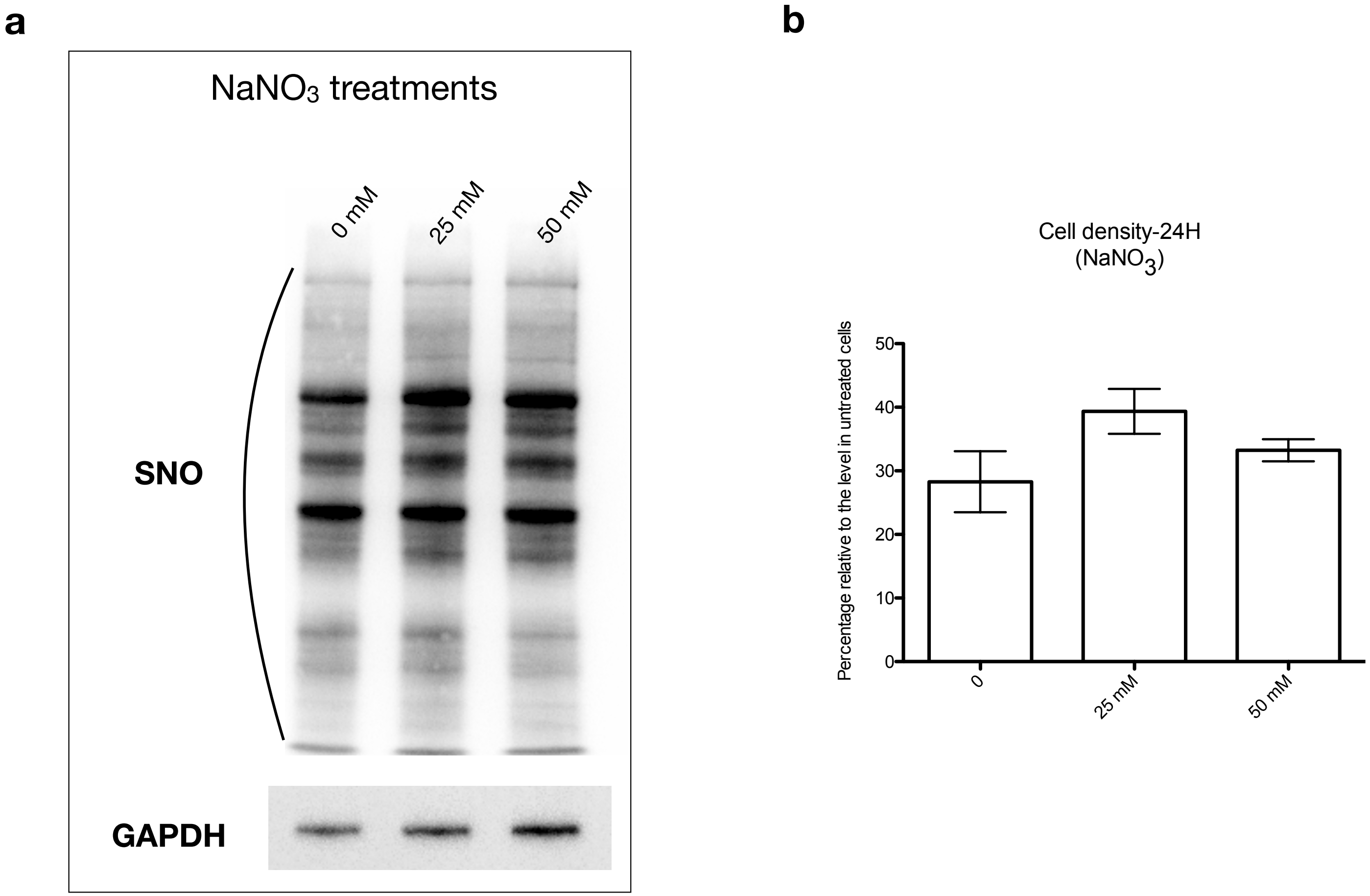

Supplement: Supplementary file 5 — Additional file 5: Figure S2. The effects of sodium nitrate treatment on ID T. vaginalis. a The SNO proteomes of ID T. vaginalis treated with 25 and 50 mM of sodium nitrate and incubated for 6 h. b The viability of sodium nitrate-treated T. vaginalis after a 24 h incubation. [file 13071_2020_4355_MOESM5_ESM.pdf]
